# Robust Prediction of Prognosis and Immunotherapeutic Response for Clear Cell Renal Cell Carcinoma Through Deep Learning Algorithm

**DOI:** 10.3389/fimmu.2022.798471

**Published:** 2022-02-07

**Authors:** Siteng Chen, Encheng Zhang, Liren Jiang, Tao Wang, Tuanjie Guo, Feng Gao, Ning Zhang, Xiang Wang, Junhua Zheng

**Affiliations:** ^1^Department of Urology, Shanghai General Hospital, Shanghai Jiao Tong University School of Medicine, Shanghai, China; ^2^Department of Pathology, Shanghai General Hospital, Shanghai Jiao Tong University School of Medicine, Shanghai, China; ^3^Department of Urology, Ruijin Hospital, Shanghai Jiao Tong University School of Medicine, Shanghai, China

**Keywords:** deep learning, renal cell carcinoma, F-box family, immunotherapy, prognosis

## Abstract

It is of great urgency to explore useful prognostic markers and develop a robust prognostic model for patients with clear-cell renal cell carcinoma (ccRCC). Three independent patient cohorts were included in this study. We applied a high-level neural network based on TensorFlow to construct the robust model by using the deep learning algorithm. The deep learning-based model (FB-risk) could perform well in predicting the survival status in the 5-year follow-up, which could also significantly distinguish the patients with high overall survival risk in three independent patient cohorts of ccRCC and a pan-cancer cohort. High FB-risk was found to be partially associated with negative regulation of the immune system. In addition, the novel phenotyping of ccRCC based on the F-box gene family could robustly stratify patients with different survival risks. The different mutation landscapes and immune characteristics were also found among different clusters. Furthermore, the novel phenotyping of ccRCC based on the F-box gene family could perform well in the robust stratification of survival and immune response in ccRCC, which might have potential for application in clinical practices.

## Introduction

Renal cancer ranks sixth in terms of incidence rate among all male malignancies, accounting for almost 5% of all male cancer patients (1). In China, there were about 74,000 new tumor cases estimated in renal in 2015 (2). Renal cancer could be classified into different pathological subtypes according to various histological features, among which clear-cell renal cell carcinoma (ccRCC) accounts for about 80% of malignant cases in renal cancer (3). Prognosis of ccRCC mainly depends on tumor characteristics, and some patients with ccRCC might suffer from quite poor prognosis with the overall survival rate less than 25% in 5 years (4). Even for patients with localized ccRCC, some of them could also be troubled by tumor recurrence after surgical intervention. Hence, it becomes necessary to find out practical prognostic markers for patients with ccRCC.

F-box proteins belong to a large family and consist of ubiquitin ligase complexes of SKP1-CUL1-F-box, which can be classified into different subtypes, including FBXL, FBXW, and FBXO (5–7). The dual roles of F-box proteins had been identified among various malignant tumors. For example, FBXW7 (F-box and WD repeat domain-containing 7) could act as a tumor-suppressor targeting various carcinogenic proteins for degradation (8). The loss function of FBXW7 could induce chromosomal instability and tumorigenesis. On the contrary, the F-box protein SKP2 (S-Phase kinase-associated protein 2) could function as a tumor oncoprotein through mediating the ubiquitylation and degradation of multiple cell cycle regulators (9). However, the prognosis value of F-box proteins in ccRCC is rarely reported by now. In addition, some uncharacterized biological functions of several F-box proteins are waiting to be explored in ccRCC. Since the F-box proteins in tumorigenesis could contribute to tumor suppression and tumorigenesis, it is interesting to perform a comprehensive analysis of F-box proteins for integrated utilization in clinical practices.

In this study, we performed deep learning-based analysis of the F-box gene family (FBG) among three independent patient cohorts to identify the important role of F-box proteins in ccRCC. Furthermore, we developed and verified FBG-related novel phenotyping of ccRCC for robust stratification of survival and immune response in ccRCC.

## Materials and Methods

### Patient Sources

We included 3 patient cohorts from The Cancer Genome Atlas (TCGA, https://portal.gdc.cancer.gov), E-MTAB-1980 (10) and Clinical Proteomic Tumor Analysis Consortium (CPTAC) (11) in this study. The included patients should be pathologically diagnosed as ccRCC and had complete follow-up information and gene expression data. After eliminating patients with incomplete information, 531 patients in the TCGA cohort, 101 patients in the E-MTAB-1980 cohort, and 98 patients in the CPTAC cohort were included for the further analysis. The basic clinicopathologic characteristics of the patients are shown in **Table S1**. In addition, the pan-cancer data of 32 types of malignancies, including normalized RNA-seq data and survival information of 10,003 patients, were also downloaded from the TCGA database.

### Robust Model Construction

**Figure 1:**
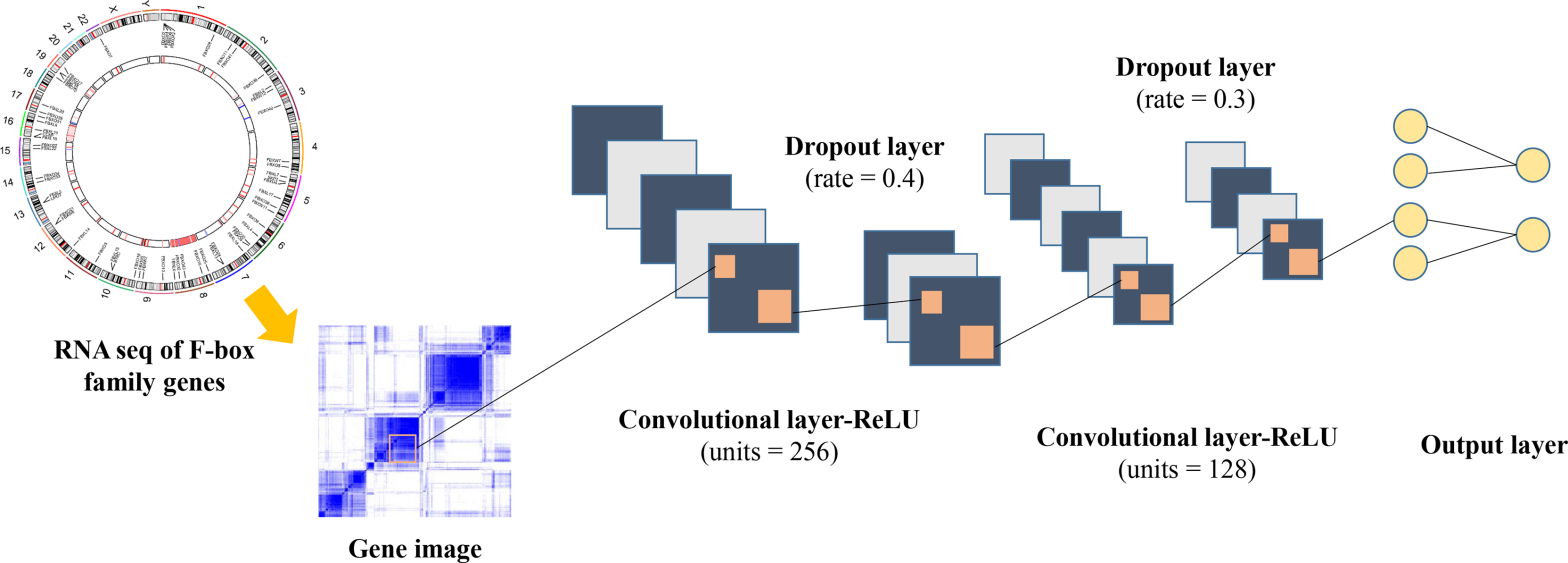
Framework of the deep neural network in this study.

In order to construct a robust model for the survival prediction of patients with ccRCC, we applied a high-level neural network (https://keras.rstudio.com) based on TensorFlow by using the deep learning algorithm. FBGs were collected according to previous reports (12–14). After eliminating genes with very low expression abundance in ccRCC, 61 FBGs were selected for further analysis (**Table S2**). The framework of the neural network is illustrated in **Figure 1**, which comprised three dense layers, with activation function of the rectified linear unit (ReLU). The model was defined using the sequential application programming interface. For the first dense layer with units of 256 and activation of ReLU, the input shape was defined as 61 since the input file contained the expressions of 61 FBGs. A dropping rate of 0.4 was set for the first dropout layer. The second dense layer was defined with units of 128 and activation of ReLU, which was followed by the second dropout layer with a dropping rate of 0.3. The loss function was set as sparse categorical cross-entropy, with an optimizer of RMSprop and metrics of accuracy.

We also applied the Least Absolute Shrinkage and Selection Operator (LASSO) (15), k-nearest neighbor (KNN) (16), XGBoost (17), and random forest (18) based on the expressions of 61 FBGs to construct machine-based prognosis prediction models for comparison (**Figure S1**). The LASSO model was carried out *via* the *glmnet* package in R to identify 7 ccRCC-related FBGs and calculate their coefficients in the TCGA cohort. The lambda value was set as 1,000 to ensure the robustness of the LASSO model. For the KNN model, we used the *psych* package in R with 5 repeats. For the XGBoost model, we used the *xgboost* package in R. The objective was set as binary logistic, and the number of rounds was set as 25. The *randomForest* package in R was used for the random forest model, and the number of trees was set as 1,000.

### Novel Phenotyping of ccRCC Based on FBG

Based on 61 FBGs in ccRCC of the C3 cluster defined by Thorsson et al. (19), we performed unsupervised cluster analysis *via* k-means (20) to explore novel phenotyping of ccRCC, in which the consensus cluster was set as 2.

### Tumor Immune Microenvironment Analysis

To identify the immune characteristics of ccRCC samples, the abundances of various immune cells for each sample were estimated through CIBERSORT (21). The expressions of chemokines, interleukins, interferons, MHC molecules, co-stimulators, coinhibitors, and other important cytokines were compared among tumors of different immune subtypes. In addition, key immune characteristics, including T-cell receptor (TCR) evenness, Th1 cells, Th2 cells, Th17 cells, cancer testis antigens (CTA) score, lymphocyte infiltration, macrophage regulation, and aneuploidy score, were also retrieved from previous research for subsequent analysis (19). The association of response to immunotherapy was also explored in a cohort of 181 patients with ccRCC, who were treated with nivolumab (anti-PD-1) and had complete clinical information (22).

### Functional Enrichment Analysis

The gene set enrichment analysis (GSEA) (23) was performed to explore potential enriched pathways, which was further visualized by *ClusterProfile* and *enrichplot* package in R (24). The number of permutations was set as 1,000. For cluster analysis, we carried out biological process analysis of gene ontology (GO) in R to find out potential enrichment pathways of each immune subtype.

### Somatic Variant Analysis

Whole-genome sequencing extracted from consensus coding mutation data was acquired from the cBioPortal (25) for somatic variant analysis. We performed the analysis *via maftools* package (26) in R, and then the different mutation landscapes of ccRCC in different immune subtypes were exhibited. Finally, the mutation statuses of the top 10 genes were visualized for each group.

### Identification of Core FBGs Associated With Response to Immunotherapy

Weighted gene co-expression network analysis (WGCNA) was carried out to develop co-expression gene networks based on FBGs *via* the *WGCNA* package in R. After identifying the module of interest, we further performed Cox regression analysis to pick out the core genes associated with the patients with ccRCC who could benefit from immunotherapy.

### Statistical Analysis

R-4.1.0 was used for statistical analysis in this study. We carried out the Mann–Whitney U test to compare continuous variables between two different groups, while variables among more than two groups were compared through one-way analysis of variance. Bonferroni was performed for adjusting the p value, and a p value less than 0.0167 was defined as significant for multiple comparisons. Kaplan–Meier curve analysis was performed to compare overall survival (OS) based on the log-rank test. The receiver operating characteristic (ROC) curve with an area under the curve (AUC) value was used for the prediction effect evaluation.

## Results

### Robust Deep Learning-Based Prognosis Prediction Model for ccRCC

**Figure 2:**
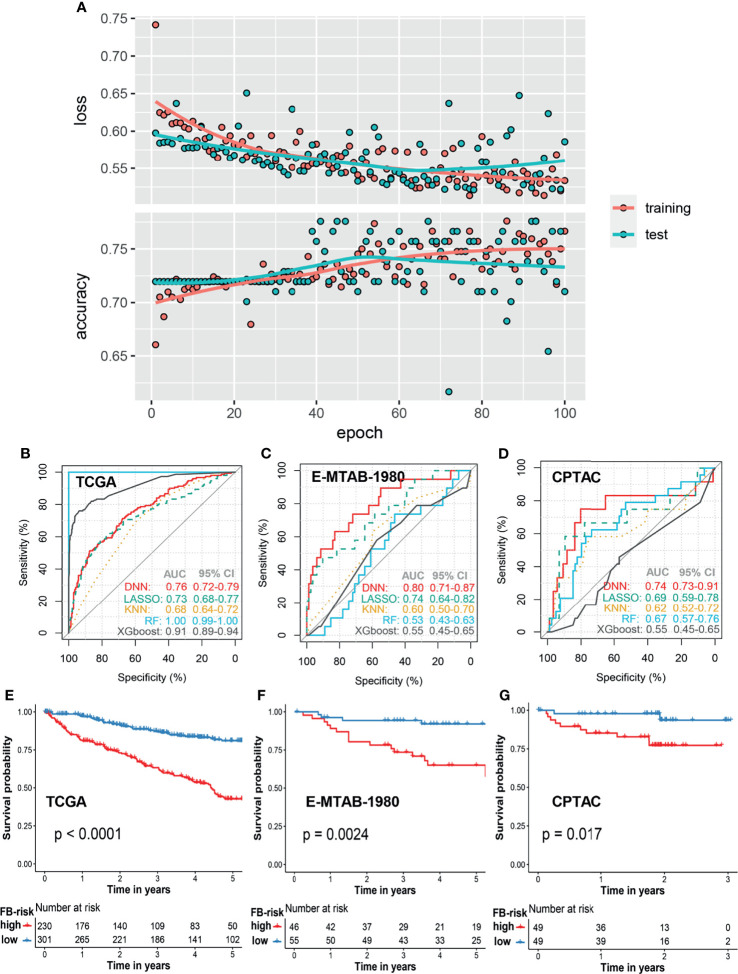
Prognosis model based on the f-box family using deep learning for ccRCC. **(A)** The learning curves of the deep learning model in the training cohort and test cohort. **(B–D)** Comparison of prognosis prediction in the 5-year follow-up using LASSO, KNN, XGBoost, RF, and deep learning methods in the TCGA cohort, E-MTAB-1980 cohort, and CPTAC cohort, respectively. **(E–G)** Kaplan–Meier survival analysis of overall survival stratified by FB-risk for ccRCC in the TCGA cohort, E-MTAB-1980 cohort, and CPTAC cohort, respectively. ccRCC, clear cell renal cell carcinoma; TCGA, The Cancer Genome Atlas; CPTAC, Clinical Proteomic Tumor Analysis Consortium; LASSO, Least Absolute Shrinkage and Selection Operator; KNN, k-nearest neighbor; RF, random forest; FB-risk, f-box family-related risk.

In order to construct a robust prognosis prediction model for ccRCC, patients in the TCGA cohort were firstly randomly divided into a training set (80%) and test set (20%). By applying the deep neural network, we trained a 5-year prognosis prediction model with an epoch of 100. The training curve in the TCGA cohort is shown in **Figure 2A**. When the cutoff value of FB-risk was set as 0.1247, the model achieved the best prediction performance in the ROC curve analysis with sensitivity of 0.71 and specificity of 0.68 (**Figure 2B**). External validations of our model were carried out in the E-MTAB-1980 cohort (**Figure 2C**) and the CPTAC cohort (**Figure 2D**). ROC curves in the E-MTAB-1980 cohort and the CPTAC cohort revealed that our deep learning-based model (FB-risk) performed better than some machine learning-based models in external validation cohorts, including LASSO, KNN, XGBoost, and random forest in predicting the survival status in a 5-year follow-up.

With the cutoff value of 0.1247, patients in the TCGA cohort and the E-MTAB-1980 cohort were grouped into a high- or low-risk group, while the cutoff value in the CPTAC cohort was set as the media value because all of the FB-risk values in this cohort were less than 0.1247, which might be due to the better prognosis of patients in this cohort. Kaplan–Meier survival analysis revealed that our FB-risk could significantly distinguish patients with high OS risk in the TCGA cohort (**Figure 2E**), the E-MTAB-1980 cohort (**Figure 2F**), and the CPTAC cohort (**Figure 2G**).

### Applying the Prognosis Model for Pan-Cancer

**Figure 3:**
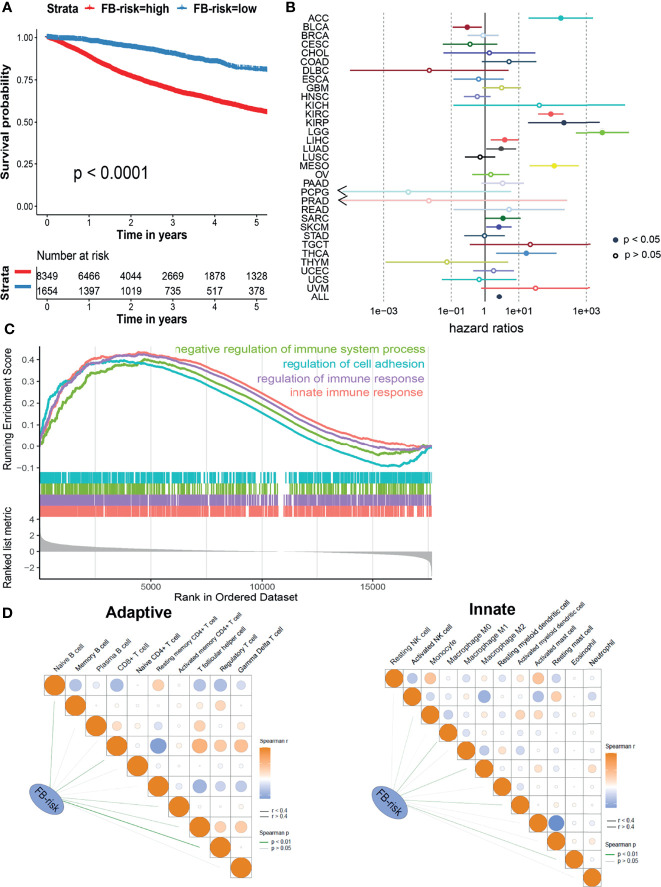
Evaluation of the prognosis model in ccRCC. **(A)** Kaplan–Meier survival analysis of overall survival stratified by FB-risk for pan-cancer patients from the TCGA dataset. **(B)** Cox regression analysis of deep learning-based FB-risk in different kinds of malignancies in the TCGA dataset. **(C)** Gene set enrichment analysis of patients with high FB-risk. **(D)** Correlation analyses of FB-risk and different adaptive immune cells (left)/innate immune cells (right) in the TCGA cohort. ccRCC, clear cell renal cell carcinoma; TCGA, The Cancer Genome Atlas; FB-risk, f-box family-related risk.

Next, we explored whether our prognosis model could be applied in the pan-cancer cohort. Based on 32 types of different malignancies from the TCGA database (10,003 patients), our model could also significantly distinguish patients with different survival risks with a cutoff value of 0.1247 (**Figure 3A**). Cox regression analysis illustrated that FB-risk could act as an independent risk factor for multiple types of tumors, including adrenocortical carcinoma (ACC), ccRCC, kidney renal papillary cell carcinoma (KIRP), brain lower-grade glioma (LGG), liver hepatocellular carcinoma (LIHC), lung adenocarcinoma (LUAD), mesothelioma (MESO), sarcoma (SARC), skin cutaneous melanoma (SKCM), and thyroid carcinoma (THCA) (**Figure 3B**), exhibiting the robust efficacy of our deep learning-based prognosis model.

### High FB-Risk Might Be Partially Associated With Negative Regulation of Immune System

In order to further explore the underlying mechanism of FB-risk, we carried out GSEA in enriched genes associated with high FB-risk (**Figure 3C**). The results showed that the genes associated with high FB-risk were mainly enriched in the negative regulation of immune system processes. The negative regulation of immune function had been widely proved to be observably associated with the progress of malignancy (27). Correlation analyses indicated that FB-risk was partially positively correlated with regulatory T cells (**Figure 3D**).

### Novel Phenotyping of ccRCC Based on f-Box Gene Family

**Figure 4:**
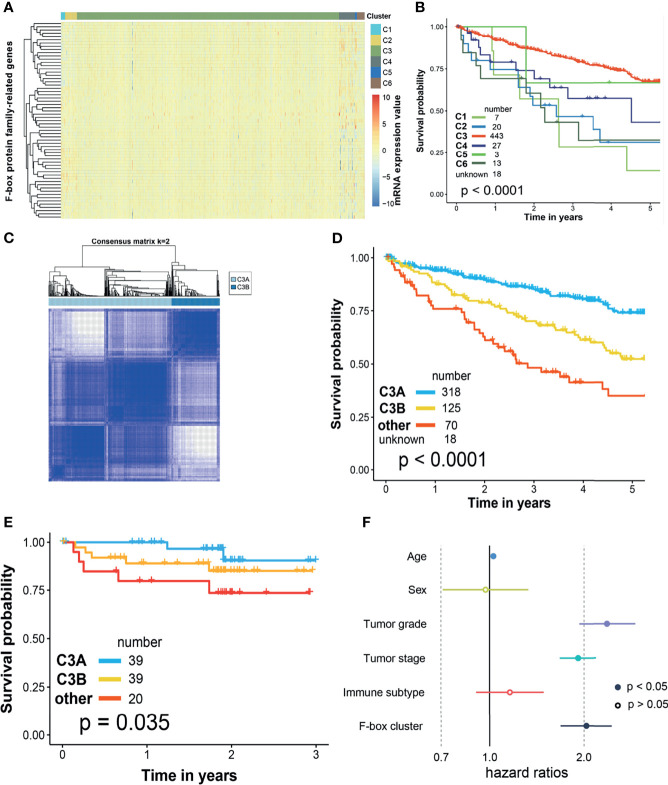
Novel phenotyping of ccRCC based on f-box family genes. Evaluation of the prognosis model in ccRCC. **(A)** Heatmap illustrated the different expressions of f-box family genes in current immune clusters in the TCGA cohort. **(B)** Kaplan–Meier survival analysis of overall survival stratified by current immune clusters FB-risk for pan-cancer patients in the TCGA cohort. **(C)** Unsupervised cluster analysis by k-means based on f-box family genes in the TCGA cohort. **(D)** Kaplan–Meier survival analysis of overall survival stratified by the novel phenotyping in the TCGA cohort. **(E)** Verification of risk stratification performance by the novel phenotyping in the CPTAC cohort. **(F)** Cox regression analysis of the novel phenotyping based on f-box cluster for ccRCC. ccRCC, clear cell renal cell carcinoma; TCGA, The Cancer Genome Atlas; CPTAC, clinical proteomic tumor analysis consortium; FB-risk, f-box family-related risk.

The current immune subtype was proposed by Thorsson et al. (19) in 2018, which was characterized by five immune signatures, namely, macrophages/monocytes, lymphocyte infiltration, TGF-β response, IFN-γ response, and wound healing. All the patients with ccRCC in the TCGA cohort could be grouped into six clusters (C1–C6, **Figure 4A**), in which there existed significantly discrepant prognosis among different clusters (**Figure 4B**). However, most of the patients (83.4%) with ccRCC were clustered into C3, which might be impractical for clinical practice. Thus, we performed unsupervised cluster analysis by k-means based on FBG to subgroup the C3 cluster. As shown in **Figure 4C**, two disparate subclusters, including C3A and C3B, could be found. When we clustered C1, C2, C4, C5, and C6 into “other cluster,” dramatically different survival outcomes could be found among patients from the C3A cluster, C3B cluster, and “other cluster” in the TCGA cohort (**Figure 4D**). Survival risk stratification by novel phenotyping was also verified in the CPTAC cohort (**Figure 4E**). Besides, Cox regression analysis revealed that the novel phenotyping system (FB-cluster) could act as an independent risk factor for ccRCC (**Figure 4F**).

**Figure 5:**
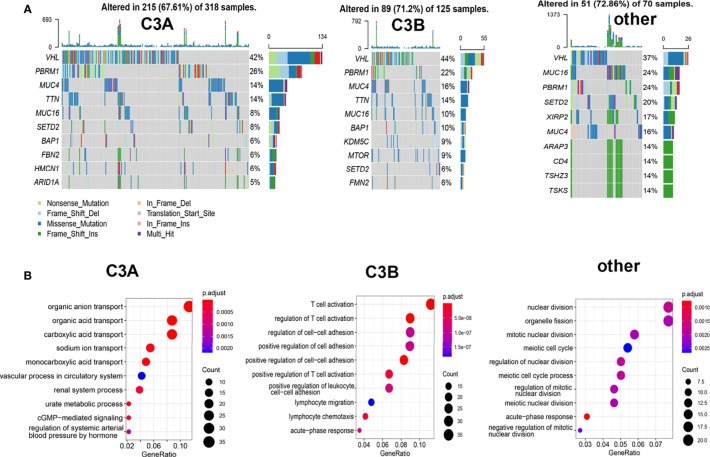
Somatic mutation and enrichment analysis of ccRCC with different immune subtypes. **(A)** Oncoplots of ccRCC in different immune subtypes. **(B)** Potentially enrichment pathways based on the enriched genes in each immune subtype of ccRCC. ccRCC, clear cell renal cell carcinoma.

Different mutation landscapes could also be observed among these three clusters. As illustrated in **Figure 5A**, a higher proportion of PBRM1-mutated tumors were found in the C3A cluster. The biological process analysis of GO indicated that the C3A cluster was memorably enriched in pathways related to metabolic transport, including organic anion transport, organic acid transport, and sodium ion transport. Meanwhile, the C3B cluster showed enrichment in the pathway of immune regulation, including regulation of T cell activation and lymphocyte migration (**Figure 5B**).

### Immune Characteristics in the C3A Cluster

**Figure 6:**
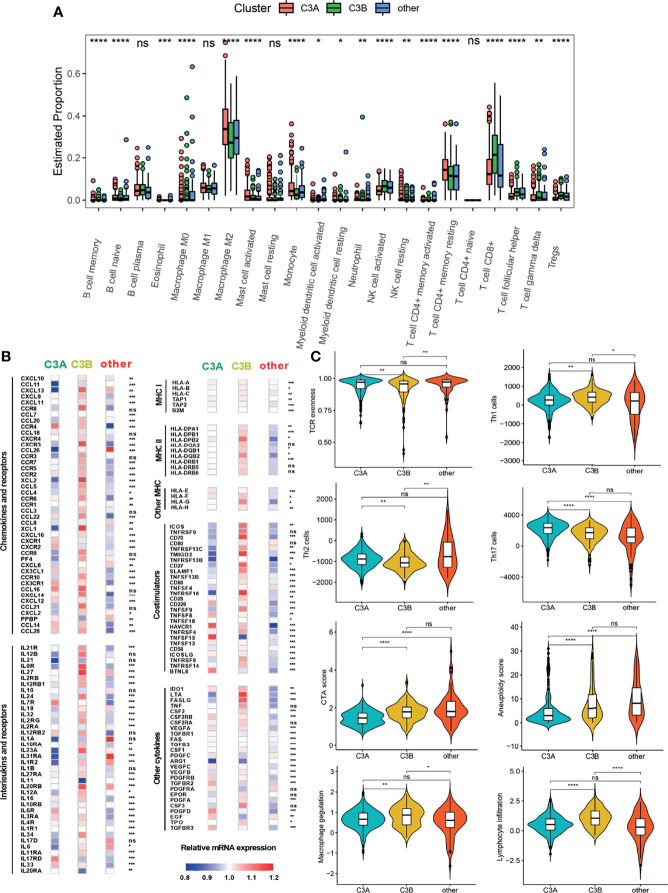
The landscape of the tumor immune microenvironment in different FB-based clusters. **(A)** Comparison of the abundances of 22 immune cells in different subclusters. **(B)** Expression of chemokines, interleukins, interferons, MHC molecules, co-stimulators, co-inhibitors, and other important cytokines and their receptors in ccRCC from different subclusters. **(C)** Different values of vital immune characteristics in different subclusters. ccRCC, clear cell renal cell carcinoma; TCR, T cell receptor; CTA, cancer testis antigens; *p < 0.05; **p < 0.01; ***p < 0.001; ****p < 0.0001; ns, non-significance.

The tumor immune microenvironment could play a vital role in tumorigenesis (28). Since patients in the C3A cluster seemed to have the most favorable prognosis, we next explored the immune characteristics of the C3A cluster. As shown in **Figure 6A**, different abundances of immune cells could be found among different clusters. The C3A cluster exhibited higher abundance of macrophage M2 and T cell CD4^+^ memory resisting cells. Immune-related genes, including CCR4, IL7R, IL17RD, HAVCR1, and TNFRSF4, were most highly expressed in C3A cluster (**Figure 6B**). In addition, ccRCC in the C3A cluster was significantly associated with higher levels of Th2 cell, Th17 cell, and TCR evenness (**Figure 6C**).

### C3A Clusters Were Associated With Increased Benefits of Immunotherapy

**Figure 7:**
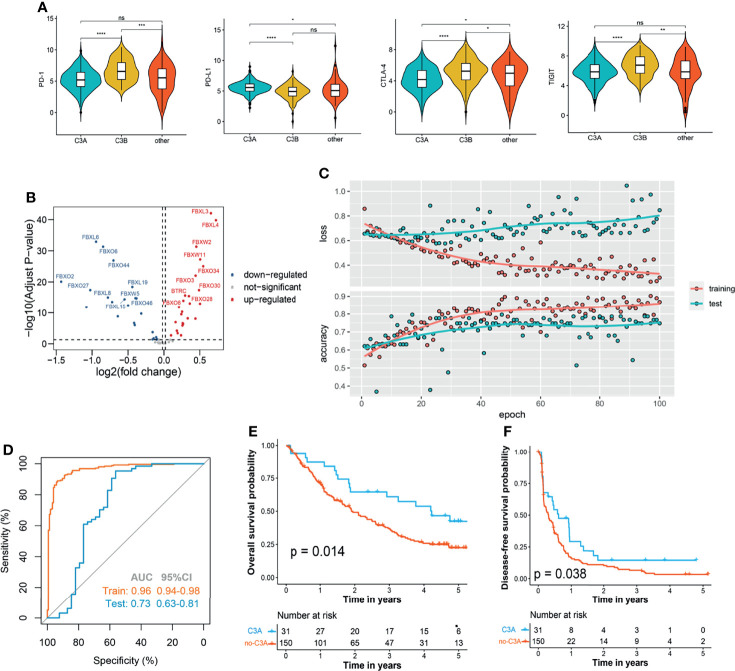
Increased response to anti-PD-1 immunotherapy exists in C3A cluster. **(A)** Comparison of the main immune checkpoints among different clusters. **(B)** Volcano plot shows the differentially expressed f-box family genes. **(C)** The learning curves of the deep learning-based model predicting the cluster of C3A. **(D)** Receiver operating characteristic curves for the prediction of C3A cluster in the training and test set. **(E)** Kaplan–Meier survival analysis of overall survival between patients treated with ICIs in the C3A cluster and others. **(F)** Kaplan–Meier survival analysis of disease-free survival between patients treated with ICIs in the C3A cluster and others. ICIs, immune checkpoint inhibitors. *p < 0.05; **p < 0.01; ***p < 0.001; ****p < 0.0001; ns, non-significance.

In the next step, we explored whether our novel phenotyping could be used for prognosis prediction of patients treated with immune checkpoint inhibitors (ICIs). Comparison of the main immune checkpoints among different clusters indicated that a higher expression level of PD-L1 was found in the C3A cluster (**Figure 7A**). In order to perform the novel phenotyping in the anti-PD-1 cohort, we firstly screened out 50 differentially expressed FBG *via* the *limma* method (29) (**Figure 7B**) and trained a C3A-cluster prediction model based on these 50 FBGs through deep neural networks (**Figures 7C, D**). The results indicated that patients in the C3A cluster exhibited observably prolonged OS and DFS (**Figures 7E, F**).

### Identification of Core FBG Associated With Response to Immunotherapy

**Figure 8:**
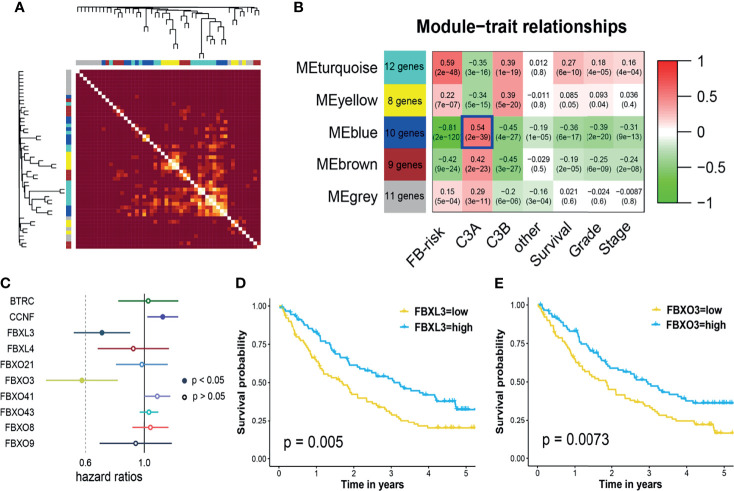
Weighted gene co-expression network and core gene analysis in ccRCC of C3A cluster. **(A)** Heatmap illustrated the different gene dendrogram based on f-box family genes. **(B)** The relationship between gene modules and clinical characteristic. **(C)** Cox regression analysis of core genes in blue modules and known immunotherapy biomarkers, including TMB, PD-1, PD-L1, and PBRM1 mutation, for patients treated with ICIs. **(D)** Kaplan–Meier survival analysis of overall survival stratified by the expression of FBXL3. **(E)** Kaplan–Meier survival analysis of overall survival stratified by the expression of FBXO3. ccRCC, clear cell renal cell carcinoma; ICIs, immune checkpoint inhibitors; TMB, total mutation burden.

All the FBGs in the TCGA cohort were hierarchically clustered into 5 gene modules through WGCNA (**Figure 8A**). Correlation analysis indicated that the blue model (MEblue) seemed to have the highest correlation with the C3A cluster (**Figure 8B**). Ten FBGs in the blue model, including BTRC, CCNF, FBXL3, FBXL4, FBXO21, FBXO3, FBXO41, FBXO43, FBXO8, and FBXO9, were then identified as hub genes. Cox regression analysis in the anti-PD-1 cohort revealed that only FBXL3, FBXO3, and PBRM1 mutation could serve as biomarkers of immunotherapy for patients with ccRCC (**Figure 8C**). Patients with higher expressions of FBXL3 and FBXO3 presented a markedly prolonged survival (**Figures 8D, E**).

## Discussion

Until recently, the most clinically applied prognostic indicators for ccRCC patients are TNM staging and the International Society of Urological Pathology (ISUP) grading system (30, 31). TNM staging mainly targets on the macroscopic features whereas the ISUP grading system concerns most on the nuclear abnormality. Under the comprehensive utilization of both TNM staging and the ISUP grading system, we found that some ccRCC patients still show unexpectedly poor prognosis, such as ccRCC patients with sarcomatous lesion and rhabdoid differentiation. Additionally, with the advent of immunotherapy for ccRCC patients, new prognostic and therapeutic biomarkers are necessary for clinical practices.

Deep learning, also known as deep neural network, can mimic the human brain to deal with complex data through multiple layers of artificial neurons (32). Considering the capability of deep learning in complex data recognition, interpretation, and generation, deep learning shows its good performance in various cancer detection and prognosis prediction based on various medical data. Based on the deep learning algorithm, we constructed and verified a robust prognosis prediction model for ccRCC, which could significantly distinguish patients with high survival risk and which performed better than traditional machine learning-based models. Traditional machine learning methods might be trapped in out-of-sample predictions due to the inconsistencies in the feature distributions and overfitting. In this study, we applied two dropout layers in the network to prevent overfitting as far as possible. The results indicated that our deep learning-based model presented better generalization performance in independent patient cohorts and a pan-cancer cohort.

It is mainly considered that F-box proteins are involved in the cell cycle regulation and the p53 apoptosis pathway to affect tumor growth (5, 6). Intriguingly, in a recent study, FBXO38 was demonstrated to mediate the ubiquitination of PD-1 on T cells and thus improve the performance of ICI treatment (33). Another F-box family member, FBXO44, could promote DNA replication and was inversely correlated with the diminished immunogenicity as well as the decreased immunotherapy response (34). In addition, F-box proteins were also reported to inhibit the metastasis of ccRCC patients and could act as prognostic indicators for patients with ccRCC (35).

As a revolutionary therapy, immunotherapy has shown its improvement in prolonging the survival of various malignant tumors, including lung cancer, gastric cancer, bladder cancer, and melanoma. Moreover, some studies illustrated that ICIs could obviously increase the survival of intermediate- or poor-risk metastatic ccRCC patients with fewer adverse reactions (36, 37). However, as a common issue for immunotherapies, only limited ccRCC patients could receive therapeutic benefits from ICIs. Therefore, it is urgent to find out some useful prognostic biomarkers for ICI application in patients with ccRCC. In this study, we proposed that the C3A cluster of ccRCC was associated with increased benefits of immunotherapy, which might have promising potential for application in clinical practices.

Further co-expression gene network and Cox regression analyses revealed that FBXL3 and FBXO3 could be the core FBGs associated with response to immunotherapy. FBXL3 was previously supposed to control the oscillation of the circadian clock (38). Recently, some studies found out that FBXL3 could also inhibit cell proliferation and migration in lung and colorectal cancer (39, 40). Moreover, FBXO3 was confirmed to regulate inflammation responses in subjects with sepsis, which was also reported to regulate ΔNp63α degradation and promote tumor metastasis in malignancy (41). Unluckily, little is known about the function of FBXL3 and FBXO3 in ccRCC for now, expressed in the regulation of response to immunotherapy. Patients with higher expressions of FBXL3 and FBXO3 presented a markedly prolonged survival in this study, illustrating that both of the two core FBGs could serve as biomarkers of immunotherapy for patients with ccRCC.

There were also some limitations in this study. Firstly, our study only included the f-box family for the construction of the prediction model. Future studies are still needed to include other known important players in ccRCC that contribute to the model performance and improve the limited accuracy. Secondly, the immunotherapy prediction model acquired high sensitivity only when the specificity was very low in the test set, which might need to be improved in further studies.

In conclusion, we proposed a deep learning-based analysis of FBG among three independent patient cohorts and identified the important role of F-box proteins in ccRCC. The robust prognosis prediction model could significantly distinguish patients with high survival risk, which also had generalization performance in multiple tumors. High FB-risk was partially associated with the negative regulation of the immune system. Furthermore, the FBG-related novel phenotyping of ccRCC performed well in the robust stratification of survival and immune response in ccRCC, which might have potential for application in clinical practices.

## Data Availability Statement

Original data for this study could be found from the TCGA data portal (https://portal.gdc.cancer.gov/) and ArrayExpress (https://www.ebi.ac.uk/arrayexpress/) under the accession number E-MTAB-1980, and Clinical Proteomic Tumor Analysis Consortium (https://proteomics.cancer.gov/).

## Ethics Statement

Ethical review and approval were not required for the study on human participants in accordance with the local legislation and institutional requirements. Written informed consent for participation was not required for this study in accordance with the national legislation and the institutional requirements.

## Author Contributions

JZ, XW, and NZ designed the study and supervised the research. JZ and NZ provided funding acquisition. SC, LJ, and EZ analyzed the data and wrote the manuscript. TG, FG, and TW collected the data. All authors contributed to the article and approved the submitted version.

## Funding

This work was supported by the National Natural Science Foundation of China (81972393 and 82002665).

## Conflict of Interest

The authors declare that the research was conducted in the absence of any commercial or financial relationships that could be construed as a potential conflict of interest.

## Publisher’s Note

All claims expressed in this article are solely those of the authors and do not necessarily represent those of their affiliated organizations, or those of the publisher, the editors and the reviewers. Any product that may be evaluated in this article, or claim that may be made by its manufacturer, is not guaranteed or endorsed by the publisher.
