# Supplementary material for: Robust Prediction of Prognosis and Immunotherapeutic Response for Clear Cell Renal Cell Carcinoma Through Deep Learning Algorithm
Source: Front Immunol. 2022 Feb 7;13:798471. doi: 10.3389/fimmu.2022.798471 (PMC8860306; doi:10.3389/fimmu.2022.798471)
Supplement: Supplementary file 1 [file DataSheet_1.docx]

**TABLE S1** Basic clinical characteristics of patients in the TCGA cohort, E-MTAB-1980 Cohort, and CPTAC cohort.

|  | **TCGA Cohort (531)** |  | **E-MTAB-1980 Cohort (101)** |  | **CPTAC Cohort (98)** |
| --- | --- | --- | --- | --- | --- |
| **Age(years)** |  |  |  |  |  |
| ≥65 | 198(37.3%) |  | 49(48.5%) |  | 41(41.8%) |
| ＜65 | 333(62.7%) |  | 52(51.5%) |  | 57(58.2%) |
| **Sex** |  |  |  |  |  |
| Male | 345(65.0%) |  | 77(76.2%) |  | 75(76.5%) |
| Female | 186(35.0%) |  | 24(23.8%) |  | 23(23.5%) |
| **Grade** |  |  |  |  |  |
| G1 | 13(2.4%) |  | 13(12.9%) |  | 6(6.1%) |
| G2 | 229(43.1%) |  | 59(58.4%) |  | 47(48.0%) |
| G3 | 205(38.6%) |  | 22(21.7%) |  | 36(36.7%) |
| G4 | 76(14.3%) |  | 5(5.0%) |  | 9(9.2%) |
| Unknown | 8(1.5%) |  | 2(2.0%) |  | 0 |
| **Stage** |  |  |  |  |  |
| I | 266(50.1%) |  | 66(65.3%) |  | 46(46.9%) |
| II | 57(10.7%) |  | 10(9.9%) |  | 11(11.2%) |
| III | 124(23.4%) |  | 13(12.9%) |  | 31(31.6%) |
| IV | 84(15.8%) |  | 12(11.9%) |  | 10(10.2%) |
| **T stage** |  |  |  |  |  |
| T1 | 271(51.0%) |  | 68(67.3%) |  | 48(49.0%) |
| T2 | 69(13.0%) |  | 11(10.9%) |  | 12(12.2%) |
| T3 | 180(33.9%) |  | 21(20.8%) |  | 37(37.8%) |
| T4 | 11(2.1%) |  | 1(1.0%) |  | 1(1.0%) |
| **N stage** |  |  |  |  |  |
| N1 | 16(3.0%) |  | 7(6.9%) |  | **/** |
| N0 | 240(45.2%) |  | 94(93.1%) |  | **/** |
| Unknown | 275(51.8%) |  | 0 |  | **/** |
| **M stage** |  |  |  |  |  |
| M1 | 79(14.9%) |  | 12(11.9%) |  | **/** |
| M0 | 422(79.5%) |  | 89(88.1%) |  | **/** |
| Unknown | 30(5.6%) |  | 0 |  | **/** |
| **Survival status** |  |  |  |  |  |
| Dead | 175(33.0%) |  | 23(22.8%) |  | 12(12.2%) |
| Living | 356(67.0%) |  | 78(77.2%) |  | 86(87.8%) |

**TABLE S2.** F-box family genes used for this study.

| **F-box family genes** | **Gene symbol** |
| --- | --- |
| Beta-transducin repeat containing E3 ubiquitin protein ligase | BTRC |
| Cyclin F | CCNF |
| F-box and leucine-rich repeat protein 12 | FBXL12 |
| F-box and leucine-rich repeat protein 13 | FBXL13 |
| F-box and leucine-rich repeat protein 14 | FBXL14 |
| F-box and leucine-rich repeat protein 15 | FBXL15 |
| F-box and leucine-rich repeat protein 16 | FBXL16 |
| F-box and leucine-rich repeat protein 17 | FBXL17 |
| F-box and leucine-rich repeat protein 18 | FBXL18 |
| F-box and leucine-rich repeat protein 19 | FBXL19 |
| F-box and leucine-rich repeat protein 2 | FBXL2 |
| F-box and leucine-rich repeat protein 20 | FBXL20 |
| F-box and leucine-rich repeat protein 22 | FBXL22 |
| F-box and leucine-rich repeat protein 3 | FBXL3 |
| F-box and leucine-rich repeat protein 4 | FBXL4 |
| F-box and leucine-rich repeat protein 6 | FBXL6 |
| F-box and leucine-rich repeat protein 7 | FBXL7 |
| F-box and leucine-rich repeat protein 8 | FBXL8 |
| F-box protein 10 | FBXO10 |
| F-box protein 11 | FBXO11 |
| F-box protein 15 | FBXO15 |
| F-box protein 16 | FBXO16 |
| F-box protein 17 | FBXO17 |
| F-box protein, helicase, 18 | FBXO18 |
| F-box protein 2 | FBXO2 |
| F-box protein 21 | FBXO21 |
| F-box protein 22 | FBXO22 |
| F-box protein 24 | FBXO24 |
| F-box protein 25 | FBXO25 |
| F-box protein 27 | FBXO27 |
| F-box protein 28 | FBXO28 |
| F-box protein 3 | FBXO3 |
| F-box protein 30 | FBXO30 |
| F-box protein 31 | FBXO31 |
| F-box protein 32 | FBXO32 |
| F-box protein 33 | FBXO33 |
| F-box protein 34 | FBXO34 |
| F-box protein 36 | FBXO36 |
| F-box protein 38 | FBXO38 |
| F-box protein 39 | FBXO39 |
| F-box protein 4 | FBXO4 |
| F-box protein 40 | FBXO40 |
| F-box protein 41 | FBXO41 |
| F-box protein 42 | FBXO42 |
| F-box protein 43 | FBXO43 |
| F-box protein 44 | FBXO44 |
| F-box protein 46 | FBXO46 |
| F-box protein 5 | FBXO5 |
| F-box protein 6 | FBXO6 |
| F-box protein 7 | FBXO7 |
| F-box protein 8 | FBXO8 |
| F-box protein 9 | FBXO9 |
| F-box and WD repeat domain containing 11 | FBXW11 |
| F-box and WD repeat domain containing 12 | FBXW12 |
| F-box and WD repeat domain containing 2 | FBXW2 |
| F-box and WD repeat domain containing 5 | FBXW5 |
| F-box and WD repeat domain containing 7, E3 ubiquitin protein ligase | FBXW7 |
| F-box and WD repeat domain containing 8 | FBXW8 |
| F-box and WD repeat domain containing 9 | FBXW9 |
| LIM domain 7 | LMO7 |
| S-phase kinase-associated protein 2, E3 ubiquitin protein ligase | SKP2 |


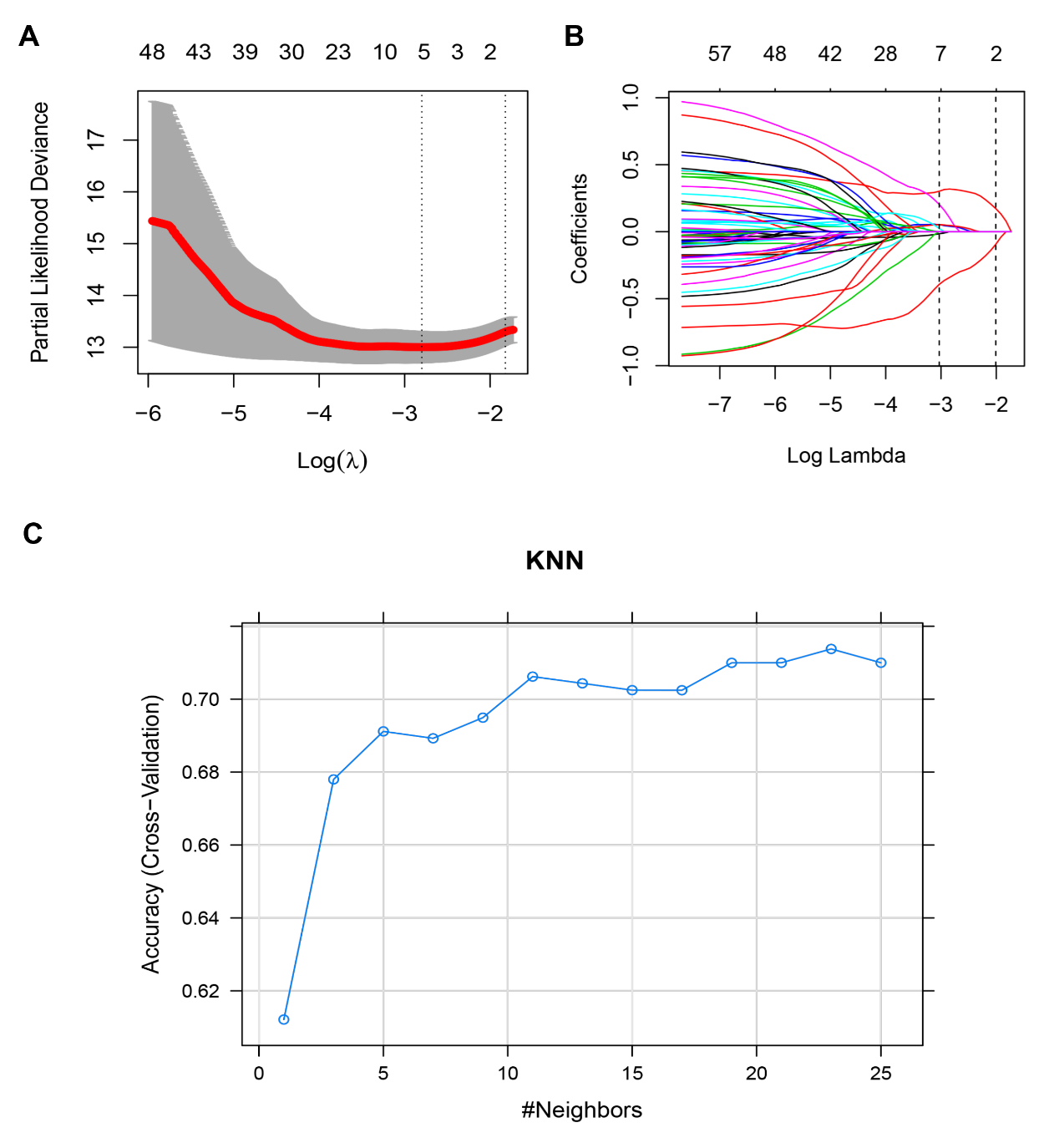


**FIGURE S1** (**A, B**) tenfold cross-validated error and coefficients at varying levels of penalization plotted against the log (lambda) sequence for the least absolute shrinkage and selection operator analysis, respectively. (**C**) Learning curve of model. KNN, k-nearest neighbor.
